# A Medical Translation Assistant for Non–English-Speaking Caregivers of Children With Special Health Care Needs: Proposal for a Scalable and Interoperable Mobile App

**DOI:** 10.2196/21038

**Published:** 2020-10-14

**Authors:** Emre Sezgin, Garey Noritz, Jeffrey Hoffman, Yungui Huang

**Affiliations:** 1 Nationwide Children's Hospital Columbus, OH United States

**Keywords:** medical translation, mobile app, special health care needs, pediatrics, caregiver-provider communication

## Abstract

**Background:**

Communication and comprehension of medical information are known barriers in health communication and equity, especially for non–English-speaking caregivers of children with special health care needs.

**Objective:**

The objective of this proposal was to develop an interoperable and scalable medical translation app for non–English-speaking caregivers to facilitate the conversation between provider and caregiver/patient.

**Methods:**

We employed user-centered and participatory design methods to understand the problems and develop a solution by engaging the stakeholder team (including caregivers, physicians, researchers, clinical informaticists, nurses, developers, nutritionists, pharmacists, and interpreters) and non–English-speaking caregiver participants.

**Results:**

Considering the lack of interpreter service accessibility and advancement in translation technology, our team will develop and test an integrated, multimodal (voice-interactive and text-based) patient portal communication and translation app to enable non–English-speaking caregivers to communicate with providers using their preferred languages. For this initial prototype, we will focus on the Spanish language and Spanish-speaking families to test technical feasibility and evaluate usability.

**Conclusions:**

Our proposal brings a unique perspective to medical translation and communication between caregiver and provider by (1) enabling voice entry and transcription in health care communications, (2) integrating with patient portals to facilitate caregiver and provider communications, and (3) adopting a translation verification model to improve accuracy of artificial intelligence–facilitated translations. Expected outcomes include improved health communications, literacy, and health equity. In addition, data points will be collected to improve autotranslation services in medical communications. We believe our proposed solution is affordable, interoperable, and scalable for health systems.

## Introduction

### Background

Children with special health care needs have a high number of hospitalizations and require more specialized services. Due to the complexity of their conditions, transitioning children with special health care needs from inpatient care to continuing therapy in the home setting is often challenging. Despite these challenges, a successful transition of care is critical to help families of children with special health care needs optimize their child’s outcome. Caregivers of children with special health care needs need to communicate with the clinical team frequently to report symptoms, request medication refills, receive care instructions, seek care advice, and discuss other health issues. Thus, ensuring that caregivers of children with special health care needs understand the discharge summaries and care plans, have timely access to communication channels to address their questions, and are given guidance to appropriate services is essential for a smooth transition of care and to achieve the best state of health for their children.

Communication and comprehension of medical information is a known barrier for patients and their caregivers, especially for non–English-speaking caregivers [1,2]. Non–English-speaking caregivers, by definition, are caregivers who have limited proficiency and understanding of the English language. Use of professional interpreters in medical communications with non–English-speaking caregivers and patients is a standard procedure. During discharge, instructions on medications and care plans are typically delivered through a professional interpreter—in person or through phone/video services [3]. However, it is possible that non–English-speaking caregivers might not fully understand the information provided during transition of care and might not feel comfortable engaging in a conversation with the clinical team. In addition, it is not common for translated medication instructions and care plans to be provided in written format, especially for less common languages. Non–English-speaking caregivers have to rely on their own notes or memories to adhere to the care or medication guidance. There are also scenarios when public health and clinical guidelines have been disseminated broadly without support for non–English-speaking communities, which may lead to inequitable care [4,5], as evidenced by the current COVID-19 pandemic crisis [6].

### Gap Analysis

Unfortunately, it can be difficult to access a certified medical interpreter whenever needed. In addition to accessibility and availability, there is the additional burden of cost to the health care system of providing interpretation services [7,8]. Another barrier to adequate interpretation for non–English-speaking caregivers is the limited engagement of interpreters at the point of care. Communication typically occurs at a single point in time and mostly focuses on specific current aspects of care without a comprehensive conversation (eg, treatment plans, assessment, diagnosis). Needing a professional interpreter creates a hurdle for patients and their caregivers outside clinical encounters, especially after hospital discharge, when non–English-speaking caregivers need information and guidance the most, including care navigation.

Under Title VI of the 1964 Civil Rights Act, public agencies are obligated to provide competent language assistance to individuals with limited proficiency in the English language. Use of certified medical interpreters is ideal, but these individuals may be limited in their availability, particularly outside of business hours, in less-resourced settings, and for less common languages or dialects. Technology-based language translation was identified as a potential approach for facilitating and improving communication among patients and health care providers [9]. Web-based translation tools, such as Google Translate, were found to be useful in patient-provider interactions [10]. However, these tools demonstrate significant quality issues in terms of discrepancy and inaccuracy for clinical communications [10-12]. Evidence suggests that significant improvements in commonly used web-based translation tools will be required before these tools can be used reliably for routine medical translation.

In summary, there is a critical need to fill this gap of translation services for non–English-speaking caregivers of children with special health care needs who need it most during transitions of care and for timely communications with providers outside of clinical settings. We propose to develop an easy-to-use mobile translation and documentation app that could be integrated with patient portals to bridge the communication gap between providers and non–English-speaking caregivers. We hypothesize that our app may reduce the dependency on medical interpreters for certain communication needs, thereby reducing potential delays in communication between non–English-speaking caregivers and their child’s care team. In addition, such an app may increase the efficiency of two-way communication and documentation of non–English-speaking caregivers by enabling message archiving into both the app and the patient’s electronic health record (EHR). In this study, our aim is to understand the technical feasibility and usability of the app for health translation services during the transition of children with special health care needs to home care.

### Prior Works

To our knowledge, there is no existing medical translation app with translation ability for Spanish users specializing in the care of children with special health care needs. Current translation services in apps (eg, Google Translate, iTranslate, and Speak & Translate) provide very limited support with generic medical term translations. All translation apps, other than Google Translate, are accessible only by paid subscribers. Currently, voice-to-text or voice-to-voice translation services for Spanish exist, but they are not integrated with medical systems [13]. Canopy Speak was found to perform well as a medical translator app [14], but it was not capable of integration with EHRs at the time of the study. Multimedia Appendix 1 provides a list of medical diary apps available on the market as of March 2020. The majority of the apps are in the English language and few support other languages. None of the diary apps have any functionality of translation or integration to EHRs for communications.

## Methods

### User-Centered Approach

The Digital Health Innovation Team and the Section of Complex Care at Nationwide Children’s Hospital (NCH) have collaborated to bring together multidisciplinary perspectives and expertise to develop digital health solutions in this area. We engaged with end users iteratively throughout the entire process of problem solving—from initial problem identification and formulation, ideation of possible solutions, integration with clinical workflow, testing of user acceptance, and choosing a deployment strategy to obtaining postimplementation user feedback.

During preliminary exploration and shadowing sessions in the clinical areas with providers, nurses, nutritionists, and pharmacists, multiple challenges of translation service availability and accessibility were identified. One example was when a mother of a patient called the clinic’s nursing line when translation services were not available. She left a message in her native language; by the time the nurse had a medical interpreter available to call the mother back, she was not available. This situation recurred multiple times during the observation period. There was consensus among the clinical team that only a few languages were common in their patient population but translation services for those languages may not be readily accessible. This leads to additional stress and burden for both families and clinical staff. In the case of urgent concerns, the lack of timely interpretation could have negative health consequences. We employed a user-centered design in first understanding the problems identified by our complex care team members, and then brainstorming together about potential solutions. Additionally, we interviewed complex care teams to better understand the clinical workflow and how potential solutions could fit into it.

After exploring the use of medical interpreters, the lack of interpreter service accessibility, and the lack of translation technology maturity, we propose a medical translation communication app, which we initially intend to support Spanish-speaking families. If successful, the app could be easily expanded to accommodate other languages. Since 2006, the staff at NCH has cared for more than 24,000 registered Spanish-speaking pediatric patients across 800,000 medical encounters and generated more than 2.8 million patient notes. We plan to interview non–English-speaking caregivers to understand their needs in medical communications, their challenges in using a traditional patient portal, and how a translation app could be seamlessly integrated into the existing channels of communication with their child’s care team. Our previous user-centered research has been successfully implemented [15], funded (eg, Challenges of the Health Resources and Service Administration’s Maternal and Child Health Bureau), and supported in complex care [16,17]. We will follow a similar strategy to engage end users and community members with our multidisciplinary stakeholder team.

### Research Team

We will build the app in-house by leveraging the expertise of our experienced developers and the information systems team at NCH. We assembled an interdisciplinary stakeholder team (including caregivers, physicians, researchers, clinical informaticists, nurses, developers, nutritionists, pharmacists, and interpreters) at our hospital to better understand and outline the existing problems and opportunities, and to create the potential solutions. Supporting this team are a number of information technology (IT) personnel who have experience building Amazon Web Services (AWS) solutions, EHR analysts, physician informaticists, other clinicians, and 150 full-time care coordinators.

### Proposed Solution

We propose to develop an EHR-integrated, multimodal (voice-interactive and text-based) communication and translation app to enable non–English-speaking caregivers and providers to communicate with one another, each in their preferred language, during transition to home care (off site). Major user functionalities are grouped into 3 categories: communication, translation, and documentation.

#### Communication

**Figure 1:**
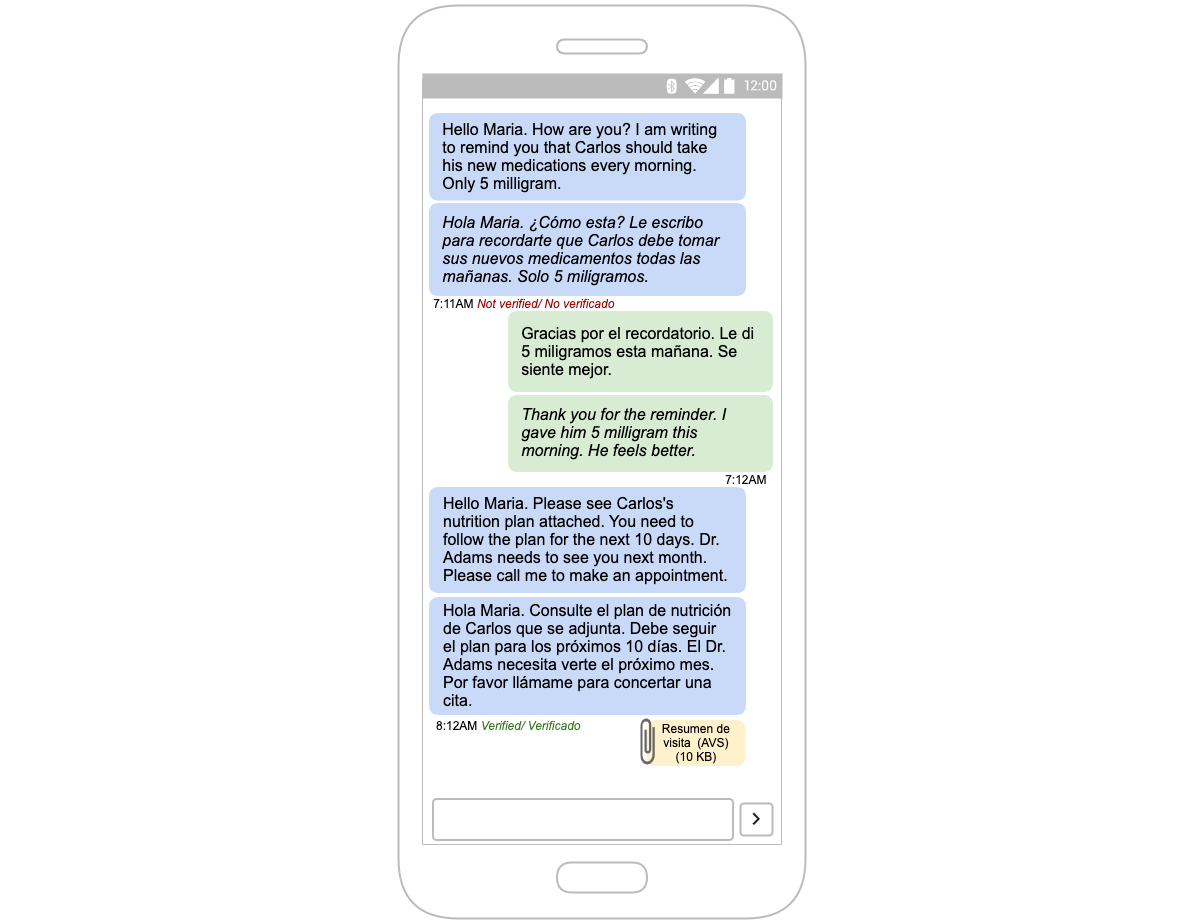
App wireframe.

Non–English-speaking caregivers will be able to enter medical messages via voice or text in Spanish. Entries will be transcribed and translated online using AWS. The provider will receive an English transcribed text (with audio attachment if voice entry is used) and will be able to respond using text or voice, which will be translated into Spanish through AWS and sent as transcribed text with an audio attachment (Figure 1).

Providers will be able to initiate communication and access communication histories through the EHR with no change to their existing clinical workflow. The app will be integrated into the EHR through the patient portal, which allows providers to see patient messages in the EHR message inbox. Providers will have access to a web app to translate their messages. The translated texts will be sent over the patient portal. Figure 1 provides an example of an app interaction between a Spanish non–English-speaking caregiver and a complex care nurse. As a nurse initiates the conversation over the EHR messaging service, it will be translated into Spanish using AWS and will appear in the app interface.

#### Translation

**Figure 2:**
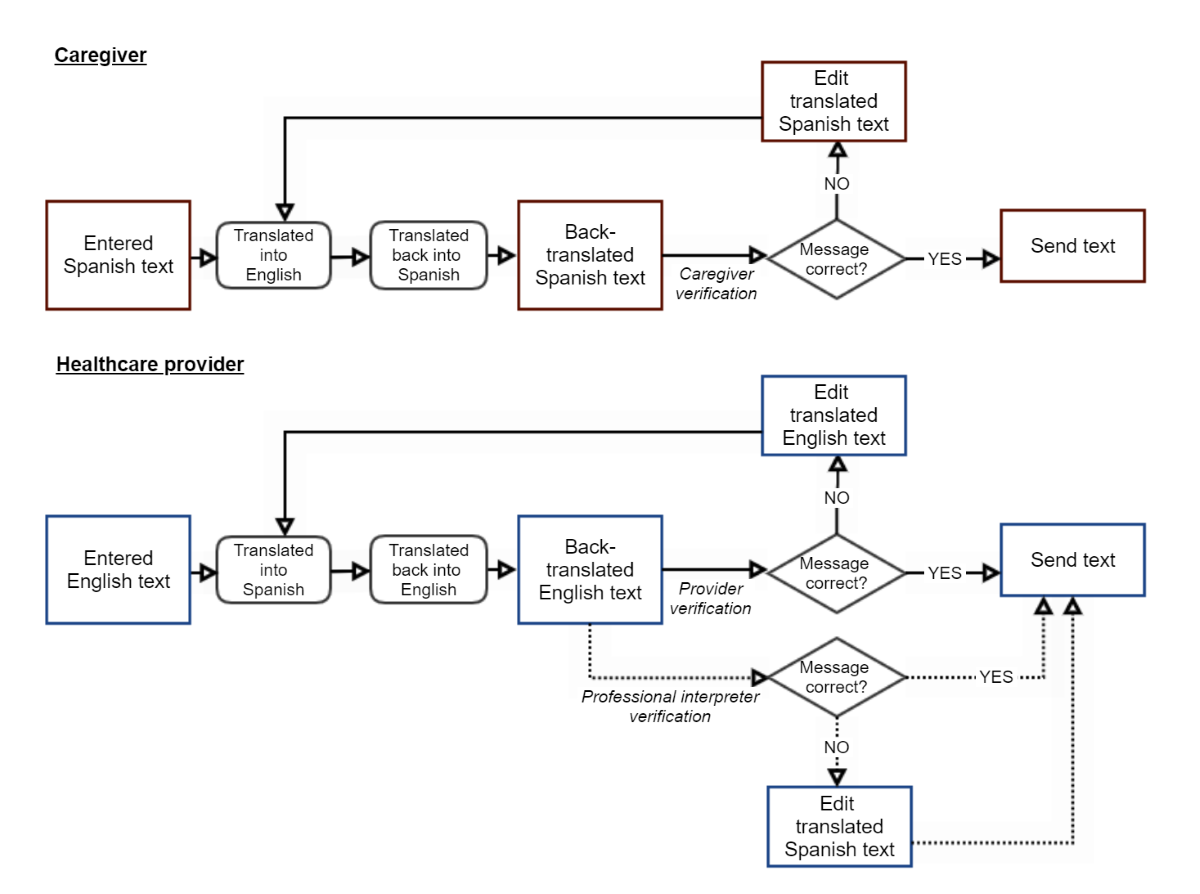
Verification process for translation.

Web-based translation services through AWS will be used to translate messages and information shared by caregivers and providers. However, to reduce the number of translation errors, we will add a verification process that requires users to manually review translated messages (Figure 2). When a caregiver enters a message in Spanish, the text will be translated into English and back into Spanish in real time. The caregiver will be able to review the Spanish text and make edits, such as rephrasing the sentence or changing words, to reduce errors. This process may continue iteratively until the caregiver is satisfied with the translation. Similarly, providers will be able to enter English text through the web app and to review translated texts.

**Figure 3:**
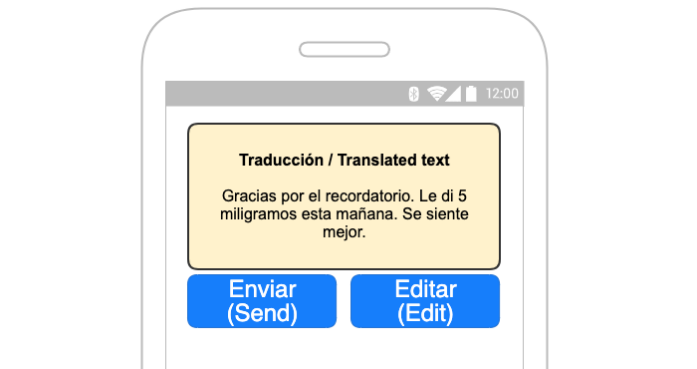
Verification screen.

When necessary, on-site professional interpreters will be able to assist with message translations by correcting and verifying complex or crucial autotranslated messages to ensure validity. Verification will be shown on the app (Figure 1 shows verified and unverified messages). Interpreter assistance will be helpful in translating discharge documents for the caregiver. Spanish non–English-speaking caregivers will also be able to use autotranslation in their communications through the app, and to correct or verify the content (Figures 2 and 3).

#### Documentation

**Figure 4:**
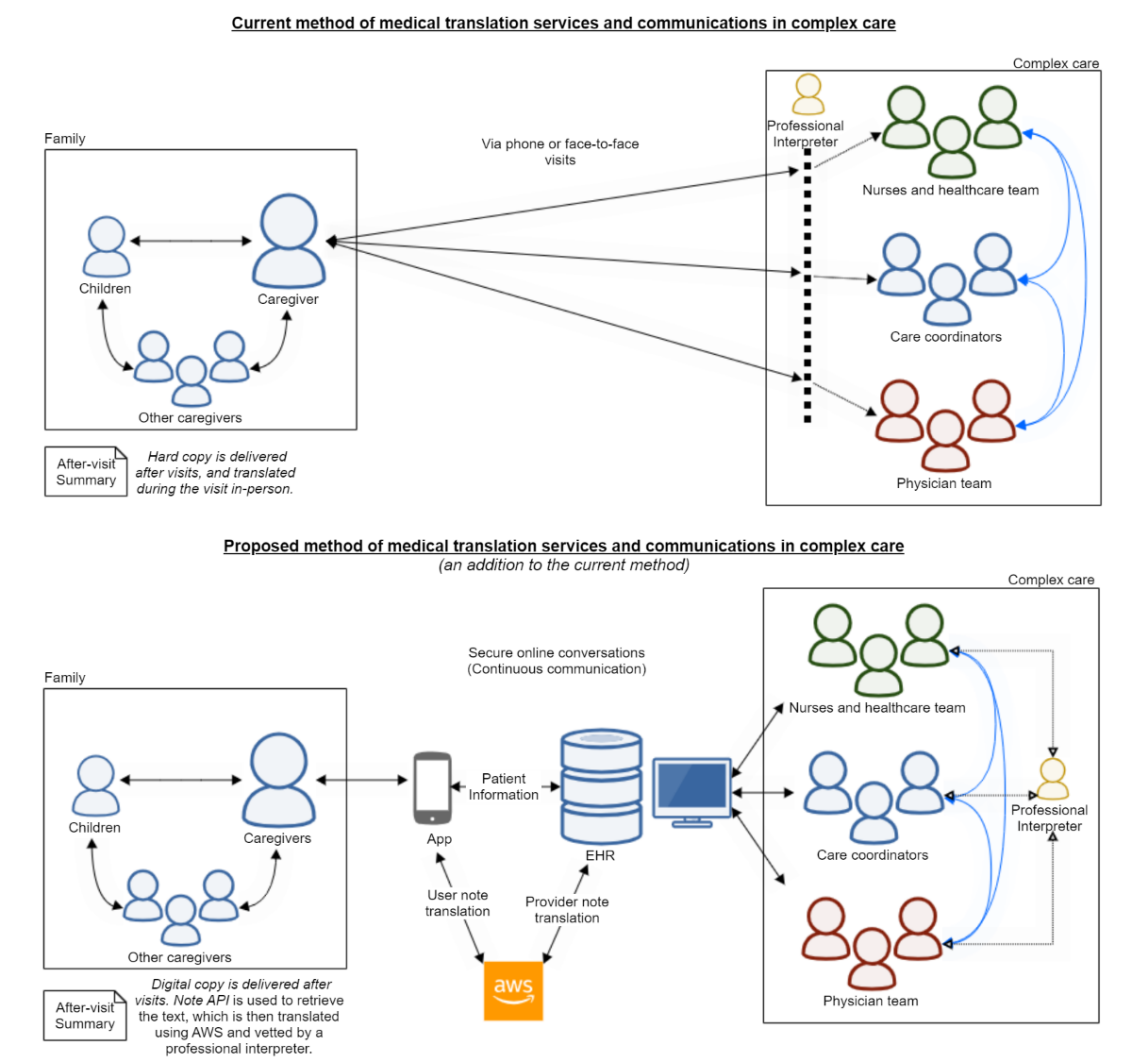
Proposed framework for medical translation services. API: application programming interface; AWS: Amazon Web Services; EHR: electronic health record.

Nurses will be able to share discharge documentation and care plans through the app, which can be translated into Spanish through AWS. The app will log each communication entry and archive it on the cloud server and in the app, which non–English-speaking caregivers can review offline. Eventually, as outlined in Figure 4, the app will take on some of the workload of medical communication and translation. In the current practice, in each medical communication during home care transition, a professional interpreter is needed to communicate transition documentation and help with phone triaging. With the proposed model, translations will be maintained by the system (translating documents and messages), and professional interpreters will be asked to help when necessary for validation of complex communications. However, in-person communications at the hospital will still be maintained by professional interpreters.

### Infrastructure and Integration

To maximize access, minimize cost, and minimize disruption to the clinical or administrative workflow, we will design a scalable, extensible, and integrated solution, leveraging AWS as our backend environment for translation services, and integrate it with our EHR system through O-Auth 2.0 and Fast Healthcare Interoperability Resources (FHIR) standards. AWS has demonstrated promising developments in translation and natural language processing services, especially with medical transcription and understanding [18], with improving accuracy and expansion to additional languages. Using AWS allows us to develop a scalable infrastructure for the app. The elasticity of AWS will enable the app to be deployable to a larger user base without any code or infrastructure changes. Integration to the EHR through FHIR is also critical for a modern health application, although traditional web services may be leveraged for specific data elements to maintain compatibility with current EHR platforms. As health care provider organizations and EHR systems increasingly support these types of interoperable standards, the IT and administrative costs have been reduced to support the integration and deployment of a mobile app that interfaces with the EHR. NCH has already established guidelines and standard processes to address security and privacy issues. Some of the technical requirements are as follows: (1) infrastructure needs of AWS’ web server, DynamoDB database, and Lex/Polly services, which may affect the cost of service as more users adopt the app, (2) AWS translation application programming interfaces between Spanish and English [19], and (3) an existing iPhone operating system (iOS) device (iOS 11+; support will be extended to Android devices in later phases).

### Evaluation

The usability and acceptance of the app will be measured using scientific methods. Our team has an extensive track record in acceptability and usability testing [17,20,21]. We will be employing a user-centered, participatory design and testing methodology, and widely adopted usability and acceptance scales, such as the system usability scale (SUS) and the technology acceptance model (TAM) [22,23]. The app will be tested by including non–English-speaking caregivers and the stakeholder team in the initial design, performing prototype and real-world testing, and eventually gathering their feedback through semi-structured interviews or surveys. Technical feasibility will be evaluated through internal assessment of the extent of interoperability with EHR and scalability with AWS.

The user-centered, participatory design protocol involves all stakeholders who will be invited to participate in design sessions. The initial meeting will aim to identify the problem space, current practice, needs, and expectations. An interactive design session is planned to capture stakeholder thoughts using words, color codes, and drawing boards. These projective tools are aimed to collect rich information and feedback from the participants [24]. Follow-up meetings will be held to communicate the initial design of the app prototype and to get feedback. The last session will be a testing session during which stakeholders and non–English-speaking caregiver participants will test the prototype.

Non–English-speaking caregiver participants (n=20) will be recruited from caregivers of NCH patients via email, phone call, text message, or face-to-face communications. Eligibility criteria are (1) limited English-language proficiency, (2) Spanish as the primary language, and (3) having a child receiving care at the complex care clinic at NCH. Non–English-speaking caregiver participants will be offered a gift card of up to US $30 for their participation. Their feedback will be collected through an electronic survey or interview that is guided by usability and acceptance questions and guidelines informed by the SUS and the TAM [22,23]. Given the limitations of face-to-face meetings during the COVID-19 pandemic, our backup plan is to complete all meetings and testing online. Our plan is to use virtual drawing boards and interaction materials to communicate needs and expectations and to receive feedback. In addition, we may run the app on a webpage for users to interact through browsers on their computers or mobile phones.

Overall, the expected participatory design and development timeline is 6 months. Throughout the study, we will periodically communicate with all stakeholders via email, SMS text messaging, or phone call to update them about development progress and get feedback when necessary. This study is exempt from institutional review board review.

## Results

Thematic analysis will be used to identify, assess, and analyze patterns in the data [25]. We will record the audio and also keep meeting notes and observational notes during the sessions. The recorded audio will be transcribed and merged into a single document with observational notes that will be kept by at least one researcher. Thematic coding will be done by following Braun and Clarke’s thematic analysis guideline: (1) familiarizing ourselves with the data, (2) generating initial codes, (3) searching for themes, (4) reviewing and refining themes, (5) defining and naming themes, and (6) reporting the findings [25]. The expected outcomes of our evaluation are qualitative input from all stakeholders and qualitative and quantitative feedback from non–English-speaking caregiver participants in response to usability and acceptability surveys. Eventually, our aim for the proposed app is to increase engagement and timely communication between caregivers and providers, which may lead to improved health outcomes [6]. A tentative timeline is shared in Table 1.

**Table 1:** Study timeline.

	Month 1	Month 2	Month 3	Month 4	Month 5	Month 6
Kickoff meeting and planning	✓					
Participatory design sessions		✓	✓		✓	
Development		✓	✓	✓	✓	
Maintenance					✓	✓
Testing					✓	✓
Reporting						✓

## Discussion

### Principal Findings

The proposed solution should lead to better health outcomes in both the short term and the long term, especially for patients with chronic and/or complex conditions, by empowering and engaging patients and caregivers. By overcoming the language barriers between clinical teams and non–English-speaking patients or caregivers, the proposed solution will transform the way they can communicate and how they work together to achieve the best outcome for the patients. By making information more accessible and communication easier, non–English-speaking patients and caregivers will (1) be equipped with the correct information in their own language, (2) be able to more actively engage in clinical care decision making, (3) be more adherent to care plans, (4) carry out needed care activities outside of clinical settings, and (5) keep the clinical team informed.

The proposed solution could also lead to better health literacy. It helps caregivers to be more familiar and comfortable with medical terminology and health information, to develop a habit to document symptoms and issues, and to improve English-language proficiency. Communicating with clinical teams and revisiting these archived communication notes would help non–English-speaking caregivers to get familiar with communication language, as well as medical terms and concepts in care communications. Currently, in-person translation services may be lacking at providing transcription of communications, which may limit non–English-speaking caregivers to learning only during the engagement. The ability to access transcripts would facilitate gaining English-language proficiency. With the support of AWS medical vocabulary and increased accuracy in capturing medical terms, transcription would include accurate medical terms (such as diagnosis and medications), and would eventually help non–English-speaking caregivers to learn the correct spelling and pronunciation of medical terms and medication names (which has also been problematic in communications, especially during phone triaging). In the long term, this engagement would have a positive impact on the effectiveness of in-person communications with providers.

In addition to improving health outcomes and literacies for individual patients and caregivers, we believe this communication transformation could lead to more equitable care for non–English-speaking communities. These communities can be hard to reach for public health announcements and dissemination of general health information due to language and cultural barriers. They have a lower rate of patient portal sign-ups and may be unable to understand health-related information posted on health care organizations’ websites. This proposed solution will encourage their adoption of patient portals, allowing them to have meaningful communications with their health care organizations. In turn, health care organizations could leverage this communication channel to disseminate important health information. In future work, we aim to propose the use of the translation service over patient portals once we gather evidence on the feasibility and performance of the app.

The proposed solution could improve the accuracy of automated medical translation services in the long term. The app will be able to collect information about original and revised translations, corrected sentences and words, frequency of translations, and used and modified texts with timestamps. On the provider side, we will be able to label original translations by professional interpreters. The data will be used to train and test natural language processing and natural language understanding algorithms to improve the online translation services for Spanish and complex care communication. In future phases of this research, this algorithm will add a layer between AWS and the app—as a quality check and correction service maintained by the research team.

The proposed solution is planned to be scalable and affordable. To patients and caregivers, the estimated cost is anticipated to be relatively low compared with conventional methods of translation, only requiring a download of a free mobile app on existing personal mobile phones and learning some intuitive functions of the app. Families may have an added expense related to their data plans. This solution requires minimal administrative, financial, clinical, and technical investments, thus offering greater accessibility. With the flexible back-end solutions, it can also be used by other patients and hospitals and is not limited to children with special health care needs or pediatric care. However, children with special health care needs and other patients with chronic illnesses may be the most motivated to adopt this tool and may benefit from it the most.

### Challenges and Limitations

One of the main limitations with online translation services is accuracy. As mentioned previously, online translation could yield unintended translations with dire consequences in a clinical setting. To avoid that, we will translate the content back to the sender to validate if the content is accurate. Figure 2 demonstrates the process and Figure 1 shows a wireframe of the app user interface for Spanish text. It will also be applicable for English text from providers in the EHR dashboard. The translation and verification process outlined in Figure 2 could take a couple of iterations if the statements are complex. To overcome this limitation, we plan to use professional interpreters to review translations on the provider side. However, non–English-speaking caregivers and providers may need to simplify communication text to increase the accuracy of translations. We aim to improve translation services in the long term as we collect more data. We plan to keep records of translations for future efforts to develop and improve artificial intelligence algorithms in language understanding and app translations in complex care. The target population is assumed to have limited English-language proficiency and adequate literacy to engage with the app. To reduce any medicolegal risk, messages sent to providers will include a notice regarding the potential risk of translation errors and a suggestion to include an interpreter service when necessary. For non–English-speaking caregivers, users will be informed about verified and unverified messages and potential errors in translations.

Further research is planned to be inclusive of low-literacy non–English-speaking caregivers through voice assistant–based engagement. The current proposal includes voice interaction in message entry only. Future implementations would include partner hospitals and organizations, as well as public and federal funding agencies to improve the solution, scale the deployment, and sustain funding. Eventually, we plan to make the app open source and to work with commercialization support offices to plan for long-term sustainability of the app.

## Appendix

Multimedia Appendix 1 Consumer-level medical diary apps.

## Abbreviations

AWS: Amazon Web Services
EHR: electronic health record
FHIR: Fast Healthcare Interoperability Resources
iOS: iPhone operating system
IT: information technology
NCH: Nationwide Children’s Hospital
SUS: system usability scale
TAM: technology acceptance model

## References

[1] Diamond LC, Schenker Y, Curry L, Bradley EH, Fernandez A (2009). Getting by: underuse of interpreters by resident physicians. J Gen Intern Med.

[2] Villarruel AM, Portillo CJ, Kane P (1999). Communicating with limited English proficiency persons: implications for nursing practice. Nurs Outlook.

[3] (2019). Using interpreter Services: Victorian Government guidelines on policy and procedures. Department of Premier and Cabinet.

[4] Cowley S, Houston A (2003). A structured health needs assessment tool: acceptability and effectiveness for health visiting. Journal of advanced nursing.

[5] Rao DV, Warburton J, Bartlett H (2006). Health and social needs of older Australians from culturally and linguistically diverse backgrounds: issues and implications. Australas J Ageing.

[6] Velasquez D, Uppal N, Perez N (2020). Equitable Access To Health Information For Non-English Speakers Amidst The Novel Coronavirus Pandemic. Health affairs.

[7] (2012). Exploring Barriers and Facilitators to the Use of Qualified Interpreters in Health. The Victorian Foundation Foundation for Survivors of Torture.

[8] O'Hagan M (2016). Massively open translation: unpacking the relationship between technology and translation in the 21st Century. International Journal of Communication.

[9] Chang DTS, Thyer IA, Hayne D, Katz DJ (2014). Using mobile technology to overcome language barriers in medicine. Ann R Coll Surg Engl.

[10] Kaliyadan F, Gopinathan Pillai S (2010). The use of Google language tools as an interpretation aid in cross-cultural doctor-patient interaction: a pilot study. Informatics in Primary Care.

[11] Patil S, Davies P (2014). Use of Google Translate in medical communication: evaluation of accuracy. BMJ.

[12] Beh THK, Canty DJ (2015). English and Mandarin translation using Google Translate software for pre-anaesthetic consultation. Anaesth Intensive Care.

[13] Panayiotou A, Gardner A, Williams S, Zucchi E, Mascitti-Meuter M, Goh AM, You E, Chong TW, Logiudice D, Lin X, Haralambous B, Batchelor F (2019). Language Translation Apps in Health Care Settings: Expert Opinion. JMIR Mhealth Uhealth.

[14] Khander A, Farag S, Chen KT (2018). Identification and Evaluation of Medical Translator Mobile Applications Using an Adapted APPLICATIONS Scoring System. Telemedicine and e-Health.

[15] Sezgin E, Weiler M, Weiler A, Lin S (2018). Proposing an Ecosystem of Digital Health Solutions for Teens With Chronic Conditions Transitioning to Self-Management and Independence: Exploratory Qualitative Study. J Med Internet Res.

[16] (2019). HRSA. 2019 HRSA MCHB Care coordination challenge winners.

[17] Sezgin E, Noritz G, Elek A, Conkol K, Rust S, Bailey M, Strouse R, Chandawarkar A, von Sadovszky V, Lin S, Huang Y (2020). Capturing At-Home Health and Care Information for Children With Medical Complexity Using Voice Interactive Technologies: Multi-Stakeholder Viewpoint. J Med Internet Res.

[18] Amazon Web Services. Amazon Transcribe Medical.

[19] Amazon Web Services. Amazon Translate.

[20] DeForte S, Sezgin E, Huefner J, Lucius S, Luna J, Satyapriya A, Malhotra P (2020). Usability of a Mobile App for Improving Literacy in Children With Hearing Impairment: Focus Group Study. JMIR Hum Factors.

[21] Militello L, Sezgin E, Huang Y, Lin S (2020). Delivering Perinatal Health Information via a Voice Interactive App: A Mixed Method Study. JMIR Preprints. 13/02/.

[22] Lewis J, Sauro J (2009). The factor structure of the system usability scale.

[23] Davis FD (1989). Perceived Usefulness, Perceived Ease of Use, and User Acceptance of Information Technology. MIS Quarterly.

[24] Sanders L, Stappers PJ (2012). Convivial design toolbox: generative research for the front end of design.

[25] Braun V, Clarke V (2006). Using thematic analysis in psychology. Qualitative Research in Psychology.

