# Supplementary material for: A Medical Translation Assistant for Non–English-Speaking Caregivers of Children With Special Health Care Needs: Proposal for a Scalable and Interoperable Mobile App
Source: JMIR Res Protoc. 2020 Oct 14;9(10):e21038. doi: 10.2196/21038 (PMC7593854; doi:10.2196/21038)
Supplement: Multimedia Appendix 1 [file resprot_v9i10e21038_app1.docx]

# Multimedia appendix 1: Consumer-level medical diary apps

This is the list of available medical diary apps in the market as of 03/2020. Majority is in English language and few supports other languages.

| **App Name** | **Features** | **Language** | **Ability to translate?** |
| --- | --- | --- | --- |
| CareClinic | - Care Plans - Used as a treatment & pill organizer - Reminders (medicine & other) - Health diary & check-ins - Track symptoms, goals, habits, etc. - Add your care-team to share reports & insights - Integration with wearables - Health planner | English | No |
|  |  |  |  |
|  |  |  |  |
|  |  |  |  |
|  |  |  |  |
|  |  |  |  |
|  |  |  |  |
| Flaredown for Chronic Illness | - Track all conditions, symptoms, and treatments in one place - Tracks thousands of chronic illnesses - Flaredown remembers your details and creates an easy check-in each day - Tag your day with details about things that may interact with your illness - Visually compare your data to test out your treatments and find triggers - Talk with other users and learn from their experience - Download your data and use it however you want (CSV format) | English | No |
|  |  |  |  |
|  |  |  |  |
|  |  |  |  |
|  |  |  |  |
|  |  |  |  |
|  |  |  |  |
| Symple Symptom Tracker | - These are the features for the free version: - Track up to 5 symptoms in just seconds per day. - Track up to 5 factors that influence how you feel. - Import steps, sleep, dietary calories & heart rate data from Apple's Health App directly into factors. - Create custom symptoms & factors or choose from our sample list. - Add one daily photo (or up to 10 with upgrade). - Share an **overview** of your symptom history with your doctor. - Export data to your favorite spreadsheet app. | English | No |
|  |  |  |  |
|  |  |  |  |
|  |  |  |  |
|  |  |  |  |
|  |  |  |  |
|  |  |  |  |
|  |  |  |  |
| The Diary | - Track efficacy of treatment and care plans - View their patient health data - Sync medical device and health app data through Apple Health - Respond to assessments and surveys - Set reminders for daily health activities - Communicate with their care team - Share with natural supports – family, friends, and caregivers - Store and share clinical records, labs, and test results | English & 11 others | No |
|  |  |  |  |
|  |  |  |  |
|  |  |  |  |
|  |  |  |  |
|  |  |  |  |
|  |  |  |  |
|  |  |  |  |
| Symptom Tracker - Health Mate | - With Symptom Tracker, family caregivers can: - Coordinate health care for parents or loved ones - Securely chat with family and doctors - Receive notifications if loved ones skip medications - Manage medical appointments for their loved ones | English & 17 others | No |
|  |  |  |  |
|  |  |  |  |
|  |  |  |  |
|  |  |  |  |
| Symdir | - Track unlimited symptoms - Record factors which you suspect influence your symptoms - View your symptoms in a easy to understand graph - Smart analyze your data, to find out what could be the course for your symptoms - Export your data and send it in a email | English | No |
|  |  |  |  |
|  |  |  |  |
|  |  |  |  |
|  |  |  |  |
